# Effective gene collection from the metatranscriptome of marine microorganisms

**DOI:** 10.1186/1471-2164-12-S3-S15

**Published:** 2011-11-30

**Authors:** Atsushi Ogura, Mengjie Lin, Yuya Shigenobu, Atushi Fujiwara, Kazuho Ikeo, Satoshi Nagai

**Affiliations:** 1 Ochadai Academic Production, Ochanomizu University, Ohtsuka 2-1-1, Bunkyo, Tokyo, 112-8610, Japan; 2 Aquatic Genomics Research Center, National Research Institute of Fisheries Science, 2-12-4 Fukuura, Kanazawa-ku, Yokohama, Kanagawa 236-8648, Japan; 3 National Institute of Genetics, Yata 1111, Mishima, Shizuoka, 411-8610, Japan; 4 National Research Institute of Fisheries and Environment of Inland Sea, 2-17-5 Maruishi, Hatsukaichi 739-0452, Japan

## Abstract

**Background:**

Metagenomic studies, accelerated by the evolution of sequencing technologies and the rapid development of genomic analysis methods, can reveal genetic diversity and biodiversity in various samples including those of uncultured or unknown species. This approach, however, cannot be used to identify active functional genes under actual environmental conditions. Metatranscriptomics, which is similar in approach to metagenomics except that it utilizes RNA samples, is a powerful tool for the transcriptomic study of environmental samples. Unlike metagenomic studies, metatranscriptomic studies have not been popular to date due to problems with reliability, repeatability, redundancy and cost performance. Here, we propose a normalized metatranscriptomic method that is suitable for the collection of genes from samples as a platform for comparative transcriptomics.

**Results:**

We constructed two libraries, one non-normalized and the other normalized library, from samples of marine microorganisms taken during daylight hours from Hiroshima bay in Japan. We sequenced 0.6M reads for each sample on a Roche GS FLX, and obtained 0.2M genes after quality control and assembly. A comparison of the two libraries showed that the number of unique genes was larger in the normalized library than in the non-normalized library. Functional analysis of genes revealed that a small number of gene groups, ribosomal RNA genes and chloroplast genes, were dominant in both libraries. Taxonomic distribution analysis of the libraries suggests that Stramenopiles form a major taxon that includes diatoms. The normalization technique thus increases unique genes, functional categories of genes, and taxonomic richness.

**Conclusions:**

Normalization of the marine metatranscriptome could be useful in increasing the number of genes collected, and in reducing redundancies among highly expressed genes. Gene collection through the normalization method was effective in providing a foundation for comparative transcriptomic analysis.

## Background

Marine microorganisms represent a major target for genetic resources and environmental monitoring [1,2]. There remain, however, many uncultured organisms so that comprehensive studies at a molecular level have long been ignored. Recently, metagenomics has been developed as a cutting-edge approach for the genomic study of marine microorganisms and other environmental samples without the need for cultivation and isolation [3]. As of May 2011, more than 470 research articles related to metagenomic studies were identified using a PubMed title search under keywords “metagenome” or “metagenomics.” Most of these studies were published within the last 5 years, indicating that this field of research has grown rapidly. This rapid growth was driven by recent developments in next-generation sequencers and high-throughput methods for genomic analysis [4,5]. A metagenomic approach has been applied to many samples, such as seawater, soil, internal organs of animal species and so on, and has revealed the species and genetic diversity in various environmental samples [6].

Metagenomics offers a valuable approach to the study of species and genetic diversity; however, this approach cannot reveal active functional genes under actual environmental conditions. Changes in the environment lead to variations in gene expression patterns in organisms, and the interactions of genes across species might change their environment. Therefore, comparative studies of metatranscriptome under various conditions or in various samples are essential to to our understanding of genetic interactions under actual environmental conditions [7-9]. However, only 18 metatranscriptomic studies had been published as of May 2011 (according to the same search procedure as for metagenome) [10-13]. Unlike genomic studies, transcriptomic data vary according to environmental conditions, and a small number of highly expressed genes can disrupt the identification of other more infrequently expressed genes [14]. Furthermore, the metatranscriptome is composed of the transcriptomes of many organisms so that, unlike single transcriptomic studies, large-scale sequencing efforts are required.

As for marine microorganism samples, we focused on plankton samples taken from the Inland Sea of Japan. Prefectural research institutes connected with Japan Fisheries have been conducting sampling of organisms for environmental monitoring in this area since the early 1970s, and have accumulated data on the appearance of phytoplankton and zooplankton [15]. Phytoplankton monitoring has shown that diatoms have been the dominant phytoplankton group (>90%) over a 35-year period, and that there was a drastic shift from Skeletonema (-70%) to Chaetoceros dominance in the mid 1980s. While the monitoring of the dominant species has been conducted and reported, there is no information available on rare species and/or smaller-sized plankton species, such as Cryptophyceae, Haptophyceae and Prasinophyceae. Very recently, a new method of plankton metagenomic analysis was developed (Nagai, in press) and this technique allows all-encompassing analyses of almost all plankton components, including zooplankton and protozoa, in coastal waters. Therefore, an integrated metagenomic and metatranscriptomic analysis will allow us to obtain detailed information on all plankton species existing in coastal waters as well as on the gene expression in each component, resulting in a more complete understanding of coastal ecosystems. For instance, metatranscriptomic analyses before and after red tides (abnormal growth of phytoplankton) may lead to the identification of the mechanisms behind red tides and the associated harmful microalgae. It may also be possible to develop a new environmental assessment technique for fishing grounds and give more scientific input to the healthy management of fishing grounds through the comparison of highly polluted and non-polluted areas.

In prior metatranscriptomic comparisons, we considered that comprehensive gene collection, even in the absence of information regarding expression frequency, would be useful in gaining a better understanding of active functional genes in samples, and would contribute to database construction and microarray design for the cost-effective monitoring of changes in gene expression in various samples. Toward an efficient gene collection method, we propose the normalization of metatranscriptome samples. Normalization, in this case, is used to reduce the interference from highly expressed genes through the use of duplex-specific nuclease [16]. We then utilize a Roche GS FLX sequencer capable of sequencing 300-500 base pairs for gene annotation. In this study, we collected a plankton sample in Hiroshima Bay (34o16′N; 132o16′E), in the Inland Sea of Japan, in December 2010. We then tested the effects of normalization using this plankton sample. We also examined the function of metatranscriptomic data and species diversity in the normalization treatment. Transcriptome data does not reflect species diversity or gene functions proportionally, but it is thought that the frequencies of expressed genes in a sample reflect the activities of functional genes in seawater.

## Results and discussion

### Comparison of normalized and non-normalized metatranscriptomic sample libraries

As noted in the Background section, one of the major purposes of metatranscriptomic analysis is to collect as many genes as possible. For this purpose, we speculated that the application of a normalization process during library construction could reduce the proportion of highly expressed genes, and contribute to the efficient collection of genes from samples. In the normalization procedure, we first denatured samples to make single-stranded DNA. We then used duplex-specific nuclease to degenerate highly expressed genes under the cooling process, whereby highly expressed genes are annealed more quickly and then digested by DNase.

To assess the efficiency of the normalization process for metatranscriptome samples, we constructed two cDNA libraries, one normalized and the other non-normalized. We utilized a Roche GS FLX system for sequencing and obtained 607,490 raw reads from the non-normalized library and 572,233 raw reads from the normalized library (Table 1). After quality control and assembly, we obtained 216,639 genes, comprising 45,064 full-length genes, 53,324 contigs and 118,251 singlets, from the non-normalized library, and 178,685 genes, comprising 49,121 full-length genes, 32,440 contigs and 97,124 singlets, from the normalized library. The smaller number of contigs in the normalized library can be explained by the lack of redundant reads that compose the contigs.

**Table 1:** Sequencing, quality control and assembly of the two libraries

		Non-normalized	Normalized
	Number of reads	607,490	572,233
Raw data	Average length	309.2bp	275.8bp
	Total base pairs	187.9Mbp	157.8Mbp

	Number of reads	483,335	373,627
Quality control	Average length	333.5bp	323.2bp
	Total base pairs	161.0Mbp	120.8Mbp

	Full-length	45,064	49,121
Assembly	Contig	53,324	32,440
	Singlet	118,251	97,124

Final	Total number of genes	216,639	178,685
	Total base pairs	73.7Mbp	57.3Mbp

Raw data was produced on a Roche GS FLX sequencer. The quality control process removed low-quality sequences and vectors. After the identification of full-length genes, the assembly process classified contigs and singlets. The total number of genes represents the sum of full-length genes, contigs, and singlets.

**Figure 1:**
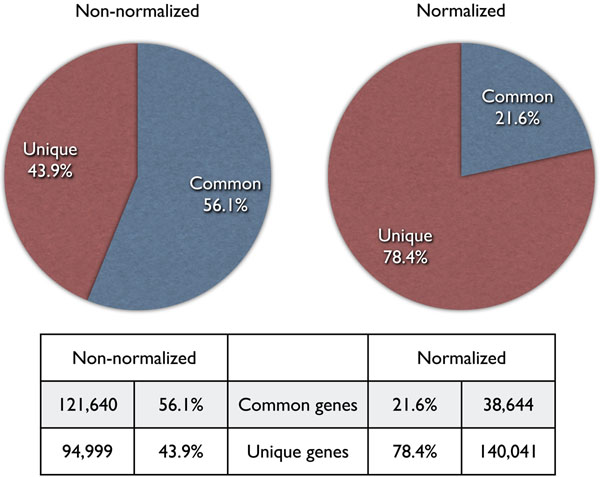
**Comparison of the two libraries and efficiency of the normalization treatment. **Reciprocal BLAT searches were performed and common genes, from non-normalized to normalized and from normalized to non-normalized, are shown as “Common.” “Unique” genes are those that do not match to each other by homology search.

To compare the two libraries, we conducted a reciprocal homology search using BLAT software with the conditions described in the Methods section. As a result, 56.1% of genes in the non-normalized library were found to have identical or highly conserved homologs in the normalized library, whereas only 21.6% of genes in the normalized library had identical or highly conserved genes in the non-normalized library (Figure 1). In other words, 43.9% and 78.4% of genes were unique in the non-normalized and normalized libraries, respectively. Normalization can, therefore, be seen to reduce redundancy among expressed genes and is suitable for the collection of various genes from marine transcriptomic samples.

Gene groups common to both libraries were thought to be highly expressed genes so we examined the frequencies of common genes in the raw data. The set of common genes consisted of 121,640 genes derived from the non-normalized library and 38,644 genes derived from the normalized library. We then counted the number of raw reads among these common genes and found that 291,487, and 171,248 reads, respectively, were included in the common gene group. This suggests that normalization treatment could reduce the number of highly expressed genes from 121,640 to 38,644 genes, or from 291,487 to 171,248 reads at the raw sequence level. We next examined the functions of common genes.

### Functional annotation of metatranscriptomic data

**Figure 2:**
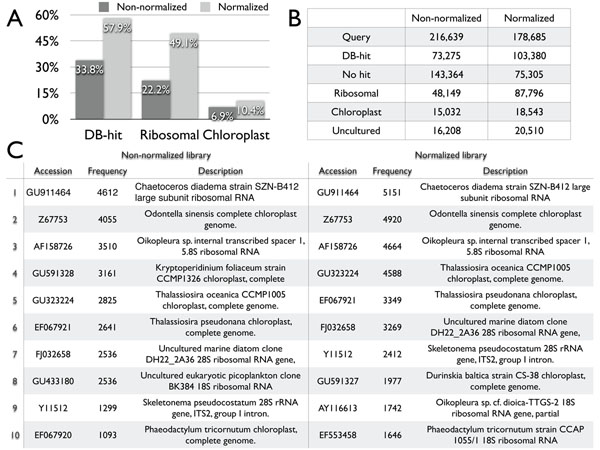
**Function of metatranscriptome data.** A. Proportions of genes hit to non-redundant nucleotide database (DB-hit), ribosomal genes, and chloroplast genes are shown in non-normalized library and normalized library. B. The numbers of total genes as query for homology search, and hit to non-redundant nucleotide database (DB-hit), no hit, ribosomal genes, chloroplast genes, and uncultured genes were shown in the table. C. Top 10 DB-hit are shown with their accession number and frequencies in the query of non-normalized library and normalized library.

The main purpose of gene collection from metatranscriptomic data is, as stated above, to collect as many genes as possible with functional annotations. For this purpose, we conducted a homology search against the nt database (non-redundant nucleotide database) taken from DDBJ. As a result, we found that 73,275 of 216,639 genes from the non-normalized library, and 103,380 of 178,685 genes from the normalized library have homologs in the DB (Figure 2). These 73,275 and 103,380 genes hit 9,307 and 9,887 genes, respectively, in the nt database. The numbers of genes hit in the database were relatively small because most genes in our libraries hit only a few genes. For example, there are many rRNA and chloroplast genes in our libraries, and it is well known that many rRNA genes are unintentionally included in the transcriptomic data [17]. We, then investigated the proportion of rRNA genes in our data, and found 48,149 of 216,639 genes (22.2%) and 87,796 of 178,685 (49.1%) genes in homology search of the non-normalized and normalized libraries, respectively (Figure 2). We also found that many chloroplast genes (15,032 and 18,543 genes, respectively) occupied 6.9 ~ 10.4% of the total gene sets (Figure 2). As our samples were taken during the day, it is reasonable that genes related to photosynthesis were active and highly expressed. These rRNA and chloroplast genes could not be removed using the SMART method during the cDNA library construction and normalization process because they are not identical and cannot be removed and digested by duplex-specific nuclease. We also found 36,718 genes regarded as genes from uncultured organisms that were submitted to databases as the result of metagenomic projects. As normalization methods cannot reduce the proportion of rRNA genes, an efficient method for removing rRNA genes is required for future metatranscriptomic analysis [18-21].

### Taxonomic distribution analysis of metatranscriptomic samples

**Figure 3:**
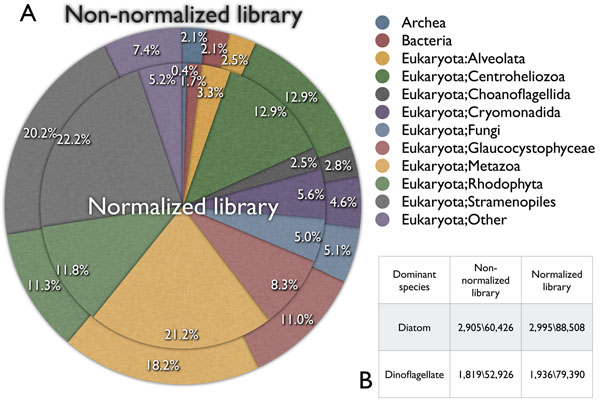
**Taxonomic distribution of the two libraries.** A. Taxonomy distribution pie-charts of the non-normalized and normalized libraries. Groups with more than 2% share are presented and all other groups are presented as “Other”. B. Genes of known dominant species, diatoms and dinoflagellates, were searched. Figures on the left represent the number of DB-hits, and those on the right represent the number of query-hits.

The taxonomic distribution of marine microorganisms is a typical focus of metagenomic studies, in which we examine the species diversity of samples [22]. In the case of metatranscriptomic studies, the distribution of genes does not imply the distribution of species. However, it remains of interest in understanding the activity of marine microorganisms. For this purpose, we undertook taxonomic distribution analysis using an rDNA database maintained at ARB, which contains all known rRNA genes with taxonomic annotation. We performed homology searches using the two libraries against the above rRNA database and obtained taxonomic distribution data (Figure 3). From this analysis, we found that the major species, at least at the level of rRNA activity, belonged to the Eukaryota domain, occupying more than 95% of the sample. This result is consistent with the fact that, in our sampling region, diatoms and dinoflagellates, which belong to the Eukaryota domain, are known to be the dominant species [15]. In fact, Stramenopiles, which include many kinds of diatoms is the major group in this analysis. We next performed the same analysis using the normalized library. As the normalization protocol reduces highly expressed gene redundancy, it is much more difficult to understand the taxonomic distribution from the data obtained. However, a comparison with the non-normalized library indicates that the reduction in the number of species in the normalized library might be due to the fact that most were major species without genetic diversity. A comparison of the two libraries further suggested that those species are often members of the Archea or Glaucocyctophycae. On the other hand, groups in which the proportions were increased in the non-normalized library, such as Metazoa, might contain various genetically diversified species. The reason why the taxonomic distribution of sequences is little changed following normalization is not evident from our results, but one possible explanation is that compression of taxonomic distribution could not achieved due to insufficient depletion of rRNA variation.

We also identified genes belonging to the dominant species in our samples; i.e., diatoms and dinoflagellates. From homology searches against taxon-specific genes taken from the NCBI taxonomy browser, we estimated diatom and dinoflagellate genes with e-values of less than 1e-20 [23-25]. As a result, we found that 60,426 and 88,508, and 52,926 and 79,390 homologous genes for diatoms and dinoflagellates in the non-normalized and normalized libraries, respectively. This result shows that the normalization technique led to a 150% increase in the richness of genes. These results are in reasonably close agreement with the report by Nishikawa, which stated that 90% of marine plankton consists of diatoms and dinoflagellates.

### Problems, solutions and future applications

An obvious problem of this normalized metatranscriptomic method is that we cannot evaluate the gene expression frequency of the sample. Based on the fact that many rRNAs genes were present in mRNA samples where they limit the opportunity to sequence infrequently expressed genes, the undertaking of metatranscriptomic studies using intact samples appears to be an inefficient and expensive strategy. Normalization in this analysis could reduce redundancy from 43% to 22%; however, many rRNA genes remained. The next target is to reduce rRNA in the library. Depletion of rRNA might allow for more efficient gene collection [18]. Once the various expressed genes have been collected in the database, we could design microarrays utilizing these genes while omitting rRNA genes. Such microarrays might be a practical solution for the metatranscriptomic study of multi-samples.

## Conclusions

Gene collection using the normalization procedure is effective in increasing the number of unique genes and in reducing the number of highly expressed genes in next-generation sequence data. Normalization appears to be effective in the identification of novel genes and the construction of gene collections without providing information on gene expression frequency. For multi-sample comparison, microarrays based on these gene collections can detect changes in gene expression and species interactions at the gene level [26].

## Methods

### Collection of seawater

A plankton sample was taken by the vertical towing of a plankton net (mesh size 20 µm) in Hiroshima Bay (34o16′N; 132o16′E) in December 2010, and the collected sample was immediately transported back to the laboratory. It was inoculated into a 50-ml centrifugation tube, and harvested by centrifugation at 1,500 x g for 2 min. The supernatant was discarded and 5 ml of the autoclaved seawater was added to disperse the plankton pellet equally. A 1-ml sample of plankton suspension was inoculated into each of four 1.5-ml tubes (A.150; Assist, Tokyo, Japan). The plankton suspension was then centrifuged at 10,000 x g for 1 min and the supernatant was completely removed by pipetting.

### mRNA extraction

For RNA extraction from the plankton pellets, we homogenized the pellets using a pellet pestle motor (Kontes Glass, Vineland, NJ, USA) for 20 s on ice, and the RNAs were extracted using an RNAqueous Kit (Ambion, Austin, Texas, USA) according to the manufacture’s protocol.

### Library construction and normalization

The normalized cDNA library was constructed as follows. We extracted poly-A RNAs from samples as described above. First-strand cDNA was normalized using Trimmer-Direct (cDNA Normalization Kit). Double-strand cDNA fractions formed by abundant transcripts were degraded by duplex-specific nuclease (DSN) and synthesized using a CDS-3M adapter and SMART IV Oligonucleotide. cDNAs were then amplified with 20 cycles of polymerase chain reaction (PCR). Amplified cDNA was quantitated using a NanoDrop system (NanoDrop Technologies, Wilmington, USA).

### Library construction for Roche GS FLX and sequencing

The normalized and non-normalized cDNA libraries were fragmented into 500-800bp using a GS FLX Titanium Rapid Library Preparation Kit (Roche) according to the manufacturer's protocol. These fragments were then amplified on beads by emulsion polymerase chain reaction, and the amplified fragments in each cDNA library were pyrosequenced on a 1/2 section of picotiterplate (one plate in total) using the 454 GS FLX Titanium system and reagents (Roche). Sequence reads were submitted to the Short Read Archive (Accession number:DRA000443).

### Quality control and assembly

We trimmed vector sequences and low-quality sequences from the raw data using the Lucy2 software developed by Li and Chou [27]. We then searched sequences with a 5' cap and poly-A tail and removed them from the subsequent assembly process as full-length sequences do not contribute to sequence assemblies. Sequence assembly was performed using the Mira3 software developed by Chevreux et al. [28].

### Homology search and databases

Homology search software, BLAT, was used to find homologous sequences between the non-normalized and normalized libraries with a threshold identity score of 100.

### Taxonomy distribution analysis

A database of fully aligned and up-to-date small (16S/18S, SSU) and large (23S/28S, LSU) subunit ribosomal RNAs taken from the SILVA databases was used to classify the taxonomic distribution of our metatranscriptomic data. We conducted a BLAT search against the above database with a cutoff score value of 100. We used Domain and Kingdom only to classify species groups, such as Eukaryota: Alveolata, already classified in the SILVA databases.

## Authors' contributions

AO and SN conceived of and designed the study. SN performed sample collection and cDNA library construction. YS and AF performed 454 sequencing of samples. ML performed quality processing and preliminary analysis using raw data produced from GS FLXs. AO and ML conducted the overall analysis using assembled sequences. AO and SN wrote the paper. All authors discussed the results and commented on the manuscript.

## Competing interests

The authors declare that they have no competing interests.

## References

[B1] Arrigo KR Marine microorganisms and global nutrient cycles Nature 2005 437 349 355 10.1038/nature04159 16163345

[B2] DeLong EF The microbial ocean from genomes to biomes Nature 2009 459 200 206 10.1038/nature08059 19444206

[B3] Patil KR Taxonomic metagenome sequence assignment with structured output models Nat Methods 2011 8 191 192 10.1038/nmeth0311-191 21358620 PMC3131843

[B4] Creer S Second-generation sequencing derived insights into the temporal biodiversity dynamics of freshwater protists Mol Ecol 2010 19 2829 2831 10.1111/j.1365-294X.2010.04670.x 20663052

[B5] Petrosino JF Highlander S Luna RA Gibbs RA Versalovic J Metagenomic pyrosequencing and microbial identification Clin Chem 2009 55 856 866 10.1373/clinchem.2008.107565 19264858 PMC2892905

[B6] Bailly J Soil eukaryotic functional diversity, a metatranscriptomic approach ISME J 2007 1 632 642 10.1038/ismej.2007.68 18043670

[B7] Tartar A Parallel metatranscriptome analyses of host and symbiont gene expression in the gut of the termite Reticulitermes flavipes Biotechnol Biofuels 2009 2 25 10.1186/1754-6834-2-25 19832970 PMC2768689

[B8] Wu J Gao W Zhang W Meldrum DR Optimization of whole-transcriptome amplification from low cell density deep-sea microbial samples for metatranscriptomic analysis J. Microbiol. Methods 2011 84 88 93 10.1016/j.mimet.2010.10.018 21044647

[B9] McGrath KC Isolation and analysis of mRNA from environmental microbial communities J. Microbiol. Methods 2008 75 172 176 10.1016/j.mimet.2008.05.019 18582973

[B10] Bomar L Maltz M Colston S Graf J Directed culturing of microorganisms using metatranscriptomics MBio 2011 2 10.1128/mBio.00012-11 PMC3069634 21467263

[B11] Gilbert JA Detection of large numbers of novel sequences in the metatranscriptomes of complex marine microbial communities PLoS ONE 2008 3 e3042 10.1371/journal.pone.0003042 18725995 PMC2518522

[B12] Gosalbes MJ Metatranscriptomic approach to analyze the functional human gut microbiota PLoS ONE 2011 6 e17447 10.1371/journal.pone.0017447 21408168 PMC3050895

[B13] Hollibaugh JT Gifford S Sharma S Bano N Moran MA Metatranscriptomic analysis of ammonia-oxidizing organisms in an estuarine bacterioplankton assemblage ISME J 2011 5 866 878 10.1038/ismej.2010.172 21085199 PMC3105763

[B14] Nolte V Contrasting seasonal niche separation between rare and abundant taxa conceals the extent of protist diversity Mol Ecol 2010 19 2908 2915 10.1111/j.1365-294X.2010.04669.x 20609083 PMC2916215

[B15] Nishikawa T Hori Y Nagai S Miyahara K Nutrient and phytoplankton dynamics in Harima-Nada, eastern Seto Inland Sea, Japan during a 35-year period from 1973 to 2007 Estuaries and Coasts 2010

[B16] Zhulidov PA Simple cDNA normalization using kamchatka crab duplex-specific nuclease Nucleic Acids Res 2004 32 e37 10.1093/nar/gnh031 14973331 PMC373426

[B17] Díez B Pedrós-Alió C Massana R Study of genetic diversity of eukaryotic picoplankton in different oceanic regions by small-subunit rRNA gene cloning and sequencing Appl. Environ. Microbiol 2001 67 2932 2941 10.1128/AEM.67.7.2932-2941.2001 11425705 PMC92964

[B18] Chen Z Duan X Ribosomal RNA depletion for massively parallel bacterial RNA-sequencing applications Methods Mol. Biol 2011 733 93 103 10.1007/978-1-61779-089-8_7 21431765

[B19] Poretsky RS Gifford S Rinta-Kanto J Vila-Costa M Moran MA Analyzing gene expression from marine microbial communities using environmental transcriptomics J Vis Exp 2009 18 24 10.3791/1086 PMC2789660 19229184

[B20] Frias-Lopez J Shi Y Tyson GW Coleman ML Schuster SC Chisholm SW Delong EF Microbial community gene expression in ocean surface waters Proc Natl Acad Sci U S A 2008 105 10 3805 10 10.1073/pnas.0708897105 18316740 PMC2268829

[B21] Gilbert JA Meyer F Schriml L Joint IR Mühling M Field D Metagenomes and metatranscriptomes from the L4 long-term coastal monitoring station in the Western English Channel Stand Genomic Sci 2010 3 2 183 93 10.4056/sigs.1202536 21304748 PMC3035373

[B22] Yarza P The All-Species Living Tree project: a 16S rRNA-based phylogenetic tree of all sequenced type strains Syst. Appl. Microbiol 2008 31 241 250 10.1016/j.syapm.2008.07.001 18692976

[B23] Bowler C The Phaeodactylum genome reveals the evolutionary history of diatom genomes Nature 2008 456 239 244 10.1038/nature07410 18923393

[B24] Gabrielsen TM Genome evolution of a tertiary dinoflagellate plastid PLoS ONE 2011 6 e19132 10.1371/journal.pone.0019132 21541332 PMC3082547

[B25] Kim S Bachvaroff TR Handy SM Delwiche CF Dynamics of actin evolution in dinoflagellates Mol Biol Evol 2011 28 1469 1480 10.1093/molbev/msq332 21149641

[B26] Ogura A Yoshida M Fukuzaki M Sese J In vitro homology search array comprehensively reveals highly conserved genes and their functional characteristics in non-sequenced species BMC Genomics 2010 11 Suppl 4 S9 10.1186/1471-2164-11-S4-S9 21143818 PMC3005928

[B27] Li S Chou H LUCY2: an interactive DNA sequence quality trimming and vector removal tool Bioinformatics 2004 20 2865 2866 10.1093/bioinformatics/bth302 15130926

[B28] Chevreux B Using the miraEST assembler for reliable and automated mRNA transcript assembly and SNP detection in sequenced ESTs Genome Res 2004 14 1147 1159 10.1101/gr.1917404 15140833 PMC419793

